# Early Detection of the Recombinant SARS-CoV-2 XAN Variant in Bulgaria: Initial Genomic Insights into Yet Another Piece of the Growing Puzzle of Recombinant Clades

**DOI:** 10.3390/microorganisms11082041

**Published:** 2023-08-09

**Authors:** Ivailo Alexiev, Ivan Ivanov, Marta Giovanetti, Eleonora Cella, Ivan Stoikov, Deyan Donchev, Lyubomira Grigorova, Anna Gancheva, Reneta Dimitrova, Fabio Scarpa, Neli Korsun, Ivelina Trifonova, Veselin Dobrinov, Todor Kantardjiev, Iva Christova, Massimo Ciccozzi

**Affiliations:** 1 National Center of Infectious and Parasitic Diseases, 1504 Sofia, Bulgaria; ivanoov@gmail.com (I.I.); ivanstoikovbt@gmail.com (I.S.); didoneobux93@abv.bg (D.D.); lyubomiragrigorova@gmail.com (L.G.); gancheva.anna@gmail.com (A.G.); naydenova.reneta@gmail.com (R.D.); neli_korsun@abv.bg (N.K.); trifonova.ivelina@abv.bg (I.T.); veselin8d8@abv.bg (V.D.); todorkantardjiev@gmail.com (T.K.); iva_christova@yahoo.com (I.C.); 2 Instituto Rene Rachou Fundação Oswaldo Cruz, Belo Horizonte 30190-009, Minas Gerais, Brazil; giovanetti.marta@gmail.com; 3 Sciences and Technologies for Sustainable Development and One Health, Università Campus Bio-Medico di Roma, 00128 Rome, Italy; 4 Burnett School of Biomedical Sciences, University of Central Florida, Orlando, FL 32816, USA; eleonora.cella@yahoo.it; 5 Department of Biomedical Sciences, University of Sassari, 07100 Sassari, Italy; 6 Unit of Medical Statistics and Molecular Epidemiology, Università Campus Bio-Medico di Roma, 00128 Rome, Italy; m.ciccozzi@unicampus.it

**Keywords:** recombinant SARS-CoV-2, XAN, BA.5, BA.2, Omicron variant, coronavirus, molecular dating, phylogenomics, COVID-19 pandemic

## Abstract

The first recombinant SARS-CoV-2 variants were identified in 2022, causing public health concerns. The importance of recombinant variants has increased especially since the WHO designated the recombinant variant XBB and its lineages as subvariants that require monitoring on 20 November 2022. In this study, we provide the first insights into the new SARS-CoV-2 variant named XAN, a recombinant composed of Omicron sub-lineages BA.2 and BA.5. To our knowledge, this is the first report on the recombinant SARS-CoV-2 XAN variant identified in Bulgaria.

## 1. Introduction

For the first time since the emergence of the COVID-19 pandemic, in January 2022, Leondios G. Kostrikis announced that his research group at the University of Cyprus in Nicosia had discovered a novel SARS-CoV-2 viral strain, which purportedly shares specific properties with the variants Delta and Omicron [1]. This was the first report of a likely recombination event occurring between two distinct SARS-CoV-2 variants, which was initially designed as a novel “Deltacron” viral strain [2]. This statement was immediately challenged by some researchers, suggesting a lab mistake might be a sounder explanation for the Cyprus laboratory findings [3]. 

Coronaviruses belong to Nidovirales, an order that has the ability to proofread their genomes during their genetic replication and recombination. Therefore, by default, it can be assumed that SARS-CoV-2 might not be capable of frequent recombination like some other viruses do, and yet has the potential for the replication of heterogeneous and dynamic populations. However, soon after the first announcement of possible recombination, SARS-CoV-2 was found to be no exception and recombination is part of its nature, which allows it to adapt and evade immunity [4]. Recombination in these viruses is possible in events involving superinfection or coinfection with different SARS-CoV-2 lineages and the first coinfection case was observed in a healthy young female patient who had sustained viral shedding [5]. Numerous recombinant lineages have been already identified in different locations and were designed by the PANGO with the ‘X-’ lineage prefix [6].

Recombinant variants, including those between Delta and Omicron, were already described, and in these viral strains, the backbone was of Delta origin, while the spike protein came from the Omicron, and thus the resulting new recombinant variant had features of both of its progenitors [7]. Moreover, the combination of variants such as Delta, which is known to cause more severe disease, and the less virulent but more contagious Omicron could lead to the emergence of more virulent and more transmittable viruses through the inheritance of the most pathogenic traits from their predecessors [7].

Recombination has been reported as one of the main driving events for the evolution of viruses and naturally, it was considered as one of the possible events that could have led to the emergence of SARS-CoV-2 itself. Already in the first few months of the pandemic, Li et al. demonstrated that the entire receptor-binding motif of the SARS-CoV-2 was introduced through recombination with coronaviruses from pangolins, an event with a key role in the evolution of SARS-CoV-2 ability to infect humans [8]. More recently, using sliding window bootstrap to highlight the regions supporting phylogenetic relationships, SARS-CoV-2 was defined as a Mosaic Genome Closely Related to Bat Viruses from Yunnan [9].

The SARS-CoV-2 Omicron variant has become the dominant circulating strain in the world as a result of its high transmissibility, as well as its capability to evade immune protection induced by both natural infection and vaccination. As a continuation of its ubiquity, intravariant recombinant lineages have emerged within the Omicron subvariants multiple times [10]. Nowadays, we are witnessing numerous recombinations in this virus, and above all, the recombinant forms seem to have a tendency to increase in number.

In this study, we provide the first genomic insights regarding the emerging SARS-CoV-2 XAN recombinant variant, which is a recombinant strain belonging to the Omicron sub-lineages BA.2 and BA.5. To our knowledge, this is the first report that provides preliminary phylogenetic insights into the SARS-CoV-2 XAN viral strain circulating in Bulgaria [11]. 

## 2. Materials and Methods

The viral RNA was isolated from 400 μL of nasal swabs suspensions obtained for routine COVID-19 diagnostic/genomic surveillance purposes of the National Center of Infectious and Parasitic Diseases, Sofia, Bulgaria, using an ExiPrep 48 Viral DNA/RNA Kit (Bioneer, Daejeon, Republic of Korea) and ExiPrep 48 Dx (Bioneer) according to the manufacturer’s instructions. Real-time polymerase chain reaction (RT-qPCR) was performed using the GeneFinder™ COVID-19 Plus RealAmp Kit (OSANG Healthcare Co., Ltd., Anyang-si, Gyeonggi-do, Republic of Korea) targeting the RdRp (RNA-dependent RNA Polymerase), E (envelope) and N (nucleocapsid) SARS-CoV-2 genes. Whole-genome next-generation sequencing (NGS) of SARS-CoV-2 was conducted on samples from randomly selected SARS-CoV-2 positive individuals by using a modified ARTIC-tailed amplicon method [12]. Briefly, after the reverse transcription step, 3 μL of the copied DNA was used in four parallel multiplex PCRs. To improve the evenness of the genome coverage, the concentrations of the ARTIC v4.1-tailed primer were normalized following the protocol developed by Benjamin Farr et al. [13]. After indexing, the libraries were purified by using HighPrep™ PCR Clean-up (MagBio Genomics Inc., Kraichtal, Germany) followed by quantification, normalization, and pooling to reach 4 nM for sequencing on an Illumina MiSeq platform with the v2 reagent kit and 500 cycles (Illumina, San Diego, CA, USA). The resulting reads were aligned trimmed, and quality filtered, the primer sequences were eliminated, and full SARS-CoV-2 genomes were assembled using Geneious Prime 2021.1 (https://www.geneious.com, accessed on 1 March 2023). 

Lineage assignment was performed on the obtained consensus sequences using the Phylogenetic Assignment of Named Global Outbreak Lineages tool (PANGOLIN) [14]. The new sequence generated in this study was compared to a diverse set of SARS-CoV-2 sequences (*n* = 3036) sampled worldwide and collected up to 15 October 2022. Considering the large amount of data available at that time, we used the Subsampler tool available at https://github.com/andersonbrito/subsampler (accessed on 15 March 2023), which in turn is a pipeline for subsampling genomic data based on epidemiological time series data. All sequences were aligned using the ViralMSA tool [15], and IQ-TREE 2.1.2 [16] was used for phylogenetic analysis using the maximum likelihood approach. The raw ML tree topology was then used to analyze and estimate the number of viral transmission events between various regions of the world. TreeTime phylodynamic analysis [17] was conducted to transform this ML tree topology into a dated tree by using a constant mean rate of 8.0 × 10^−4^ nucleotide substitutions per site per year, after the exclusion of outlier sequences. The mutation pattern of the VOC was analyzed using the NextClade online tool [18]. In addition, we used data from the Global Lineage Surveillance (COVID GC) https://covidcg.org/ (accessed on 30 March 2023) to further analyze trends in the geographic distribution of all recombinant SARS-CoV-2 variants up to the time of writing this article, March 2023 [19]. See Table 1 for the detailed list of XAN mutations.

## 3. Results

A total of 22,434 samples from patients with SARS-CoV-2 were sequenced until October 2022 in Bulgaria. The Bulgarian SARS-CoV-2 XAN clade was isolated from a 76-year-old woman who spent four days in a clinic and was discharged after her recovery.

**Figure 1:**
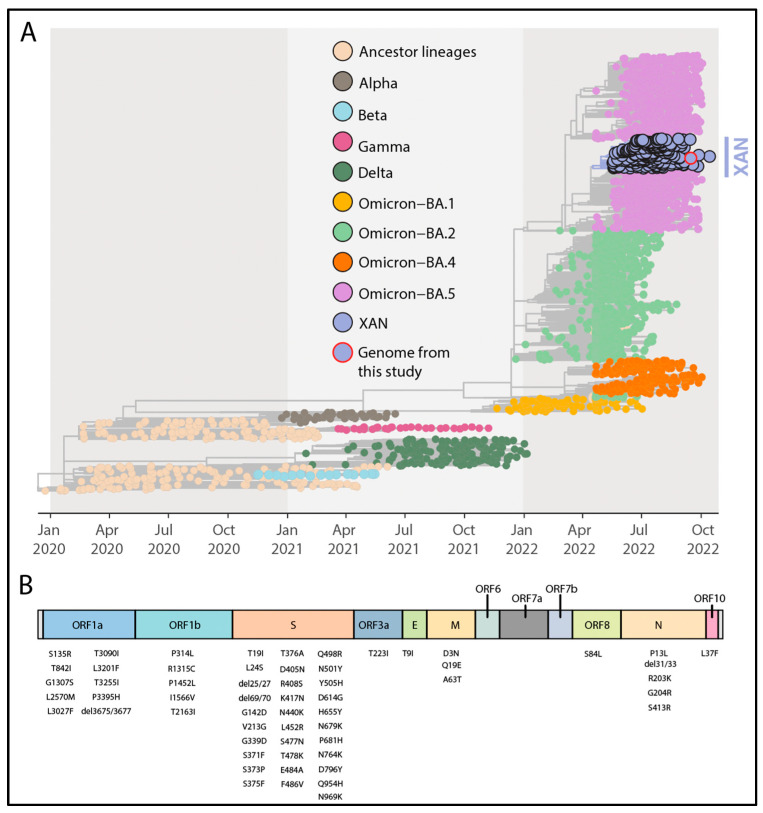
Phylogenetic analysis and genomic map of SARS-CoV-2 XAN. (**A**). Time-resolved maximum-likelihood tree of SARS-CoV-2 including a representative worldwide subsample of genomes (*n* = 3036) collected up to 15 October 2022. The genomes are colored according to the lineages (VOC and ancestral lineages) as the legend on the top left and the genome generated in this study have been highlighted in the tree (purple fill with a red circle). (**B**). Genome map of the XAN lineage characteristic substitutions. Each gene is labeled, and the substitutions are placed at the bottom (see Table 1 for the detailed list of mutations).

Lineage assessment was conducted using PANGOLIN (available at https://github.com/hCoV-2019/pangolin, accessed on 15 March 2023) and revealed that the new strain belonged to the SARS-CoV-2 XAN lineage. Phylogenetic inference by combining our new isolate (EPI_ISL_15390336) with a representative dataset available on GISAID (https://www.gisaid.org/, accessed on 1 March 2023) up to 15 October 2022 revealed that the newly obtained genome belongs to the XAN lineage and clustered closely with SARS-CoV-2 XAN strains isolated in Europe, North America, and the Caribbean between May and September 2022 (Figure 1A) (bootstrap = 1.0, SH-aLTR = 1.0). Further, we analyzed the specific mutational profile of the newly generated strain to determine its lineage-defining mutations. The newly identified lineage harbored 58 substitutions that are characteristic of XAN with substitutions interspersed across the viral genome (Figure 1B).

XAN has a BA.5-like mutational profile as fifty-five of the substitutions highlighted are shared with BA.5 sub-lineages. For the remaining three substitutions, one is unique to the XAN lineage (ORF1a:L2570M), whereas ORF1a:L3201F and ORF1b:P1452L are shared with BA.2 lineages and C.36.3.1, respectively. 

**Figure 2:**
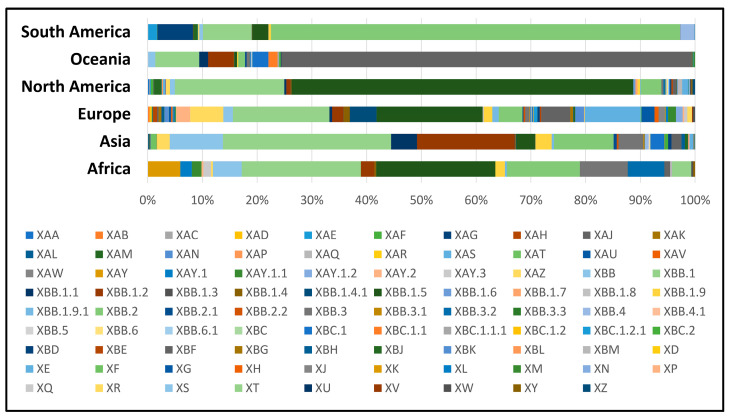
A variety of 89 different recombinant (X-) sub-lineages of SARS-CoV-2 (January 2022–February 2023). Different sub-lineages are unevenly distributed in different geographical regions.

**Figure 3:**
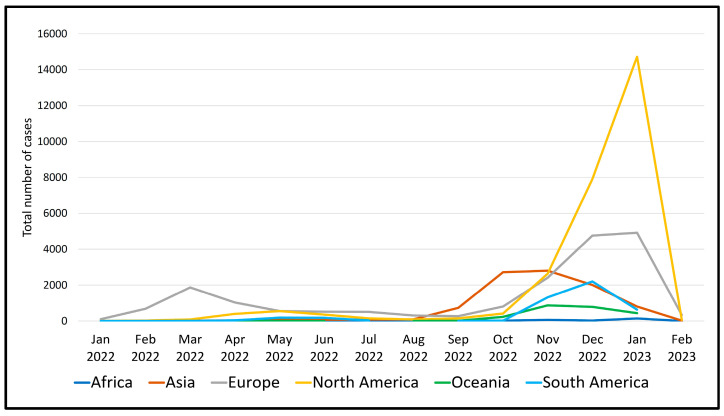
Total number of recombinant SARS-CoV-2 cases (January 2022-February 2023) in the different geographic regions.

As of February 2023, a significant number of 63,361 sequences from a total of 89 different recombinant (X-) sub-lineages of SARS-CoV-2 could be found in the COVID GC databases (Figure 2). Their diversity is considerable, with unequal geographical distribution across different continents. The number of cases caused by recombinant viruses steadily increased in 2022 and after October, it rose sharply and peaked in January 2023, especially in North America, followed by a decline in February 2023 (Figure 3).

The complete dataset used for the analysis will be provided upon request to the principal investigator.

## 4. Discussion

Bulgaria was substantially affected by the pandemic with high mortality rates against the background of low vaccination coverage [20]. After the introduction and distribution of the less virulent Omicron and its sub-variants, the mortality rate in the country significantly decreased.

To date, at least 27 different recombinant XA subvariants have been reported (XA to XAZ). Among these, the most prevalent were XAZ (35%) followed by XAM (9.8%) and XAY.2 (9.5%). A specific regional distribution could be observed, with XAZ being more prevalent in Asia and Europe, while XAM is found almost exclusively in North America. The largest proportion of new cases with XA sub-lineages has been registered from May to September 2022 and almost disappeared afterwards. Notably, XAY.2 a Delta–Omicron recombinant variant that emerged in 2022 continues to circulate efficiently in the European population, with a significant number of sequences reported in December 2022–January 2023. Currently, the WHO’s Tracking SARS-CoV-2 variant system has identified eight different variants of increased importance to public health. Six of them have been identified as recombinant viruses, including XBB.1.5 and XBB.1.16, which are listed as variants of interest (VOIs), and XBB*, XBB.1.9.1, XBB.1.9.2 and XBB.2.3 listed as variants under monitoring (VUMs).

Despite its significant mutational background inherited from two branches of Omicron BA.2 and BA.5, the transmission rate and overall spread of XAN remained limited, with only 216 sequences that have been deposited in GISAID (up to 09.01.2023) [21]. Almost 85% of the isolates were reported from European countries (*n* = 183), whereas in the US, it seemed to be less frequent (*n* = 12). The first XAN sequences were first reported from Spain and Switzerland in May 2022 and later in Denmark and Greece. The highest morbidity peaks and the corresponding estimated daily proportions for XAN were registered during July and September 2022, while in November, it already abated [21].

Further analysis of the clinical course of cases infected with XAN is necessary to perform a comprehensive assessment of the severity and mortality of the disease, as well as the efficacy of the vaccine protection in this variant. However, compared to other recombinant variants (e.g., XBB), XAN appeared less virulent but still better adapted than other XA sub-lineages that emerged at the same time. XAN, as the other early recombinant subvariant, appears as an intermediate step in the ongoing evolution of SARS-CoV-2, highlighting its exceptional plasticity [22].

It is known that the evolution of viruses, including recombinations, could impact their accurate diagnosis through substitutions in the viral genome targeted by the PCR tests. The genomic sequences of XAN inherited from BA.2 and BA.5 and the composition of mutations characteristic of XAN do not seem to negatively affect standardized multiplex PCR tests. However, the continuous evolution of viruses also requires continuous adaptation of the tests, for diagnostics and sequencing [23]. Our findings have some limitations and despite the significant number of sequenced samples of over twenty-two thousand until October 2022, not all cases of COVID-19 were referred for sequencing, because they did not meet the participant selection criteria or because they had an insufficiently high viral load required for the sequencing analysis. Further research on the identified SARS-CoV-2 XAN variant and the associated epidemiological and clinical data could broaden the spectrum of knowledge to better understand the evolution of these viruses.

Finally, our results can supplement those from other studies to expand and update our knowledge for the development of more efficient vaccine prevention and treatment of the disease.

In conclusion, consistent genomic surveillance of circulating variants remains of high importance for the early detection and monitoring of emerging SARS-CoV-2 viral strains including recombinant viruses. Therefore, monitoring the SARS-CoV-2 evolution over time remains a priority to adapt our defenses against the pandemic (see [24,25]).

## Figures and Tables

**Table 1:** List of mutations of XAN for all genes compared to the wild type (Wuhan-1 lineage). Sites listed in the table indicate the difference in genomic composition with the wild type. Sites not indicated present the same amino acid to wild type. In red bold font are the mutations of interest.

ORF1a	ORF1b	S	ORF3a	E	M	ORF8a	N	ORF10
S135R	P314L	T19I	T223I	T9I	D3N	S84L	P13L	L37F
T842I	R1315C	L24S			Q19E		DEL31-33	
G1307S	P1452L	DEL25-27			A63T		R203K	
L2570M	I1566V	DEL69-70					G204R	
L3027F	T2163I	G142D					S413R	
T3090I		V213G						
L3201F		G339D						
T3255I		S371F						
P3395H		S373P						
DEL3675-3677		S375F						
		T376A						
		D405N						
		R408S						
		**K417N**						
		N440K						
		**L452R**						
		**S477N**						
		T478K						
		E484A						
		F486V						
		Q498R						
		**N501Y**						
		Y505H						
		D614G						
		H655Y						
		N679K						
		**P681H**						
		N764K						
		D796Y						
		Q954H						
		N969K						

## Data Availability

The Bulgarian SARS-CoV-2 sequence used for the study has been deposited in GISAID under accession numbers: EPI_ISL_15390336.
